# Built-in redundant killing by Bt Cry1Ab toxin delays insect resistance

**DOI:** 10.1073/pnas.2507583122

**Published:** 2025-06-09

**Authors:** David G. Heckel

**Affiliations:** ^a^Max Planck Institute for Chemical Ecology, Department of Entomology, Jena D-07745, Germany

One of the great success stories of agricultural biotechnology is crop protection from pest insects using transgenic plants producing insecticidal proteins from the bacterium *Bacillus thuringiensis* (Bt). The benefits due to reduced application of chemical insecticides and increased yield have overcome initial concerns about the safety of transgenic technology, and Bt crops were planted to 109 million hectares worldwide by 2019 (1). Yet the evolution of Bt resistance in insect pests is increasing and threatens the sustainability of these benefits. Maize producing the Cry1F toxin was withdrawn from the market in Puerto Rico due to sudden and intractable resistance in a major corn pest, the fall armyworm (2). In PNAS, Wang et al. (3) describe mechanisms of resistance to Cry1F and the related Cry1Ab toxin in the Asian corn borer (ACB), *Ostrinia furnacalis*, in China. The results have important implications for the sustainability of Cry1Ab-expressing maize in controlling the European corn borer (ECB), *Ostrinia nubilalis* in North America.

The Cry proteins are pore-forming toxins produced by various strains of Bt to aid the bacteria in killing and growing on insects that happen to consume bacterial spores loaded with these crystalline proteins. Expression of the toxin genes in maize and cotton has vastly increased the selection pressure on insect pest populations to evolve resistance. After the pest caterpillar consumes spores or transgenic crops, the toxin is processed by proteases in the digestive system, where it binds to receptors in the midgut epithelial membrane. As a result of these binding interactions with cadherins and ABC transporters, Cry toxin monomers associate into a tetrameric “prepore” which inserts into the epithelial cell membrane, forming a cation-selective pore. This usually kills the epithelial cells, causing cessation of feeding and eventual death of the insect. Receptor mutations that block this process are generally recessive, providing the rationale for the “high dose/refuge” strategy to delay the increase of resistance in pest populations, by masking recessive mutations with susceptible alleles from insects dispersing from nontransgenic crops (4). Another effective resistance management strategy is termed “stacking” or “pyramiding”: the expression of two toxins with different receptor types in the same plant. Mutations in the receptor for one of the toxins will not affect the “redundant killing” potential of the other (5).

**Fig. 1.**
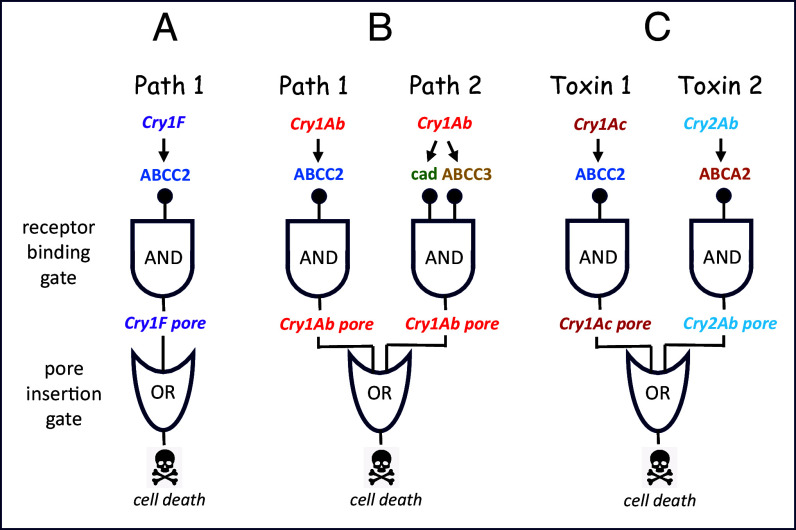
Effects of Cry toxins on cell death, as depicted by logic circuit diagrams. (*A*) Cry1F toxin binding to the ABCC2 receptor of ACB results in formation of a Cry1F pore (output of the degenerate AND-gate) which when inserted in the membrane, causes cell death (output of the degenerate OR-gate); Path 1. (*B*) Cry1Ab toxin can also interact with ABCC2 in order to form a Cry1Ab pore as output from the first AND-gate (Path 1). Alternatively, Cry1Ab must interact with both cadherin and ABCC3 to form a Cry1Ab pore as output from the second AND-gate (Path 2). Either pore disrupts membrane integrity, so the output of the OR-gate is cell death. (*C*) Illustration of redundant killing of the cotton bollworm by Cry1Ac and Cry2Aa toxins produced by Bollgard® II cotton. Cry1Ac interacts with its receptor ABCC2, forming a Cry1Ac pore from the first AND-gate. Cry2Aa interacts with its receptor, a different ABC transporter ABCA2, forming a Cry2Aa pore from the second AND-gate. Either pore may insert into the membrane, resulting in cell death depicted by the OR-gate.

The study of Wang et al. (3) is a technological tour-de-force, combining the complementary methods of CRISPR/Cas9 to knock out receptors of Cry1F and Cry1Ac in vivo, with heterologous expression of different combinations of receptors in insect cells and frog oocytes in vitro. The results with Cry1F are summarized in Fig. 1*A*, where I have borrowed some symbols from circuit theory. In Path 1, Cry1F interacts with receptor ABCC2 to form a pore as represented by the degenerate AND-gate; insertion of the pore into the membrane produces cell death as shown by the degenerate OR-gate. ABCC2 knockout strains were up to 7,500-fold resistant to Cry1F. In the more complicated case of Cry1Ab (Fig. 1*B*), the toxin must interact with either ABCC2 (Path 1) or with both cadherin and ABCC3 (Path 2), represented by two AND-gates (knockouts were up to 9,300-fold resistant to Cry1Ab). The output of each of the two AND-gates creates a pore, either one of which causes cell death when inserted into the membrane—the output of the OR-gate.

Fig. 1*C* depicts the classical “stacking” strategy of expressing two toxins with different modes of action in the same plant. Cry1Ac and Cry2Ab are expressed in Bollgard® II cotton for control of the cotton bollworm (6). The Cry1Ac interacts with its receptor ABCC2 to produce a pore, the Cry2Ab interacts with its different receptor ABCA2 to form a different pore, and either pore results in cell death. Here, the OR-gate represents the resistance management principle of redundant killing. The evolution of resistance is delayed because it takes longer for resistance-causing mutations in two different targets to appear in the population.

In PNAS, Wang et al. (3) describe mechanisms of resistance to Cry1F and the related Cry1Ab toxin in the Asian corn borer (ACB), *Ostrinia furnacalis*, in China.

This comparison suggests an explanation for the remarkable effectiveness and durability of transgenic maize expressing Cry1Ab in the Corn Belt of the midwestern United States of America, with no ECB resistance reported in the last 25 y. ECB populations have been suppressed to such low levels that even conventional, nontransgenic maize (7) and neighboring vegetable crops (8) enjoy the “halo effect” of reduced ECB damage. The characteristic damage of cornstalks caused by corn borers is now so rare that demonstration plots with conventional maize artificially infested with ECB larvae are set up to educate growers who have never seen such damage (9). If results from ACB can be extrapolated to ECB, the single toxin Cry1Ab may actually have a built-in redundant killing mechanism, due to the two different paths that can produce a lethal pore and kill the midgut cells via the OR-gate.

This remarkable durability is now endangered by the recent observation of ECB-caused damage to Cry1F-expressing sweet corn in Canada (10), likely due to mutations in ABCC2, i.e., Path 1 (11). A recent survey estimated resistance ratios to Cry1F of up to 41 and resistance ratios to Cry1Ab of up to 3.2 in Canadian populations (12). These incipient resistance levels are low compared to the ACB knockouts, but as ABCC2 mutations spread southward, new resistance-conferring mutations in the cadherin or ABCC3 threaten to erode the effectiveness of the built-in “redundant killing” of Cry1Ab. A mutation in either target could knock out Path 2, an important vulnerability of the second AND-gate. Experimental CRISPR/Cas9 knockouts in ECB are urgently needed to see how heterozygous or homozygous mutants would be selected for by Cry1Ab-expressing maize, and whether they would be selected against in nontransgenic maize, which would boost the effectiveness of the high-dose/refuge strategy. These mutations should also be evaluated for their interaction with other selective pressures on ECB populations, for example, maize defensive chemicals such as kauralexins and benzoxazinoids (13) or maize proteases that digest the caterpillar peritrophic matrix (14).

Field experiments and modeling are also needed to estimate how soon a traveling wave of resistant ABCC2 alleles could reach the midwestern United States of America, given the two major reproductive barriers to gene flow in ECB. The so-called E and Z strains are partly reproductively isolated due to different sex pheromones, and univoltine strains with one generation per year are isolated from multivoltine strains due to asynchrony in adult mating. Erik B. Dopman and colleagues have studied how the coupling effect of these two barriers to gene flow in ECB enhances reproductive isolation beyond their individual effects (15). Thomas W. Sappington has characterized the movement ecology of adult ECB with respect to managing resistance to Bt maize (16).

Additional details about the AND-gates and OR-gates need to be worked out before we know how far the results on ACB are applicable to other systems, such as the corn earworm which is evolving resistance to Cry1Ab maize in the United States of America (17). Ping Wang has argued for the existence of separate paths of toxicity in the soybean looper *Trichoplusia ni* (18), and although I initially contested this view (19), the new results of Wang et al. (3) confirm the generality of the concept. Moreover, recent work from another group in China has shown the same two pathways for Cry1Ac toxicity for the oriental armyworm *Mythimna separata* (20).

A final point to be made is the vulnerability of international scientific collaboration in the current political climate in the United States of America (21). The research described by Wang et al. (3) resulted from a collaboration between a group in China (Nanjing Agricultural University) and the most influential exponent of Bt resistance management (University of Arizona). This research in turn owes a great deal to developments from around the world over many years. This is as it should be: insecticide resistance is a global problem, and global efforts will be needed to successfully combat it.
